# Pain Experience and Patient‐Reported Barriers to Analgesic Use in Emergency Care in Ghana: A Cross‐Sectional Study

**DOI:** 10.1002/hsr2.72135

**Published:** 2026-03-19

**Authors:** Stephen Mensah Arhin, Isaac Tabiri Henneh, Joseph Arhin, James Kojo Prah, Emmanuel Nkrumah, Oluwayemisi Esther Ekor, Kwesi Boadu Mensah, Martins Ekor

**Affiliations:** ^1^ Department of Pharmacology School of Medical Sciences University of Cape Coast, Cape Coast Ghana; ^2^ Department of Pharmacotherapeutics and Pharmacy Practice School of Pharmacy University of Cape Coast, Cape Coast Ghana; ^3^ Department of Internal Medicine Cape Coast Teaching Hospital Cape Coast Ghana; ^4^ Department of Community Medicine, School of Medical Sciences University of Cape Coast Cape Coast Ghana; ^5^ Department of Acute, Continuing and Chronic Care, School of Nursing The University of Alabama at Birmingham Alabama USA; ^6^ Department of Anaesthesia and Pain Management, School of Medical Sciences University of Cape Coast Cape Coast Ghana; ^7^ Department of Pharmacology, School of Pharmacy and Pharmaceutical Sciences Kwame Nkrumah University of Science and Technology Kumasi Ghana

**Keywords:** analgesics, barriers, emergency department, Ghana, pain management, patient beliefs

## Abstract

**Background and Aims:**

Effective pain management remains a critical component of emergency care. However, patient‐related beliefs and misconceptions about pain medication can pose significant barriers to adequate analgesia. The aim of the study was to assess patient‐reported barriers to pain medication use and outcome in an emergency setting in Ghana.

**Methods:**

A cross‐sectional study was conducted among 386 adult patients who presented with moderate to severe pain. Data was collected using an adapted short form of Barriers Questionnaire, and Numerical Pain Rating Scale (NPRS) scores before and 30 min after treatment. Data was analyzed using descriptive statistics, Chi‐square test, and ANCOVA. Pain severity was categorized as mild (0–3), moderate (4–6), and severe (7–10).

**Results:**

Most participants (69.4%) demonstrated moderate to high levels of perceived barriers to pain management. The most endorsed barrier was the belief that pain signifies worsening illness (mean = 3.77, SD = 1.32), followed by concerns about addiction and saving medications for future use. Significant reductions in pain scores were observed after treatment across analgesic groups. After adjustment for baseline pain and diagnosis, differences in post‐treatment pain was significant.

**Conclusions:**

Patient misconceptions about pain and its treatment persist in emergency care settings and may impact both pain reporting and medication adherence. Efforts to improve pain outcomes must include patient education and revision of prescribing practices, especially regarding opioids and non‐steroidal anti‐inflammatory drugs (NSAIDs). Targeted interventions are essential to overcoming both systemic and attitudinal barriers to pain management at emergency settings.

## Summary

- A substantial proportion of emergency department patients reported moderate to high perceived barriers to pain medication use, highlighting persistent patient‐level challenges to optimal acute pain management.
- Sociodemographic factors, such as gender, occupation, and education level were significantly associated with perceived barriers, indicating that patient beliefs and expectations toward analgesics differ across population subgroups.
- The findings highlight the need for patient‐centered education and context‐specific pain management strategies to improve analgesic acceptance and treatment outcomes in emergency care settings in resource‐limited settings.

## 1 Introduction

Pain is one of the most common and distressing symptoms experienced by patients presenting to emergency departments (EDs) worldwide. Effective pain management is not only a clinical necessity but also a fundamental human right and a core component of quality healthcare [1, 2, 3].

However, evidence consistently shows that pain remains undertreated in many emergency care settings, particularly in low‐ and middle‐income countries (LMICs), including Ghana [4]. Inadequate pain relief can result in delayed recovery, emotional distress, and decreased patient satisfaction with care [5, 6, 7].

Patient‐related barriers, such as attitudes and anxieties, are crucial causes of inadequate pain management [8, 9]. These barriers include fear of addiction, beliefs that pain medications should be reserved for severe pain, or that expressing pain may distract healthcare providers from treating the underlying illness [8, 10]. The presence of such beliefs may lead patients to delay requesting pain relief or to underutilize prescribed medications.

The Barriers Questionnaire (BQ), developed by Ward 1993, has been widely used to explore these attitudinal obstacles in diverse clinical contexts [9]. While most existing studies have focused on cancer patients or chronic pain populations [11, 12], relatively little is known about these barriers in acute pain settings, such as emergency departments, where pain is often sudden. Moreover, sociocultural and systemic differences in many LMICs may uniquely shape patient beliefs about pain and pain relief, underscoring the need for context‐specific research [13, 14, 15]. In order to improve clinical practice, guide focused therapies, and eventually raise the standard of pain management in emergency medical settings, it is essential to comprehend these dynamics. This study therefore aimed to examine patient‐reported barriers to pain medication use and outcome in an emergency setting in Ghana, and how pain management outcomes relate to the type of analgesic prescribed.

## 2 Methods

### 2.1 Study Design and Setting

This was a descriptive cross‐sectional study conducted at the emergency department of the University of Cape Coast Hospital, Cape Coast, Ghana, over a 3‐month period (August–November 2025).

### 2.2 Population and Sampling

Eligible participants were adults (≥ 18 years) who presented with acute pain (≥ 4/10 on numeric pain scale) and were conscious and able to consent. The sample size was estimated using the Cochran Formula [16]. A total of 386 patients were recruited using consecutive sampling.

### 2.3 Sample Size Calculation

The required sample size was calculated to estimate the proportion of patients reporting barriers to analgesic use with 95% confidence and a 5% margin of error. Assuming maximum variability (50% prevalence), the minimum sample size was 384. After adjusting for the finite population and a 10% non‐response allowance, the final target was approximately 373 participants. We enrolled 386 participants, exceeding this target.

### 2.4 Questionnaire

The Barriers Questionnaire‐II (BQ‐II) [9] was adapted for local use and pre‐tested. We made use of a modified 8‐item instrument scored on a 0–5 Likert scale (0 = do not agree at all; 5 = agree very much). The study questionnaire was adapted from the short‐form Barriers Questionnaire. Eight of the 12 original items addressing core barrier domains and applicable to the emergency context were retained to improve relevance and reduce respondent burden. Additional sections were included to capture sociodemographic information, clinical diagnosis/indications, type of analgesic administered, and numerical pain rating scores before and after treatment. The adapted questionnaire was pre‐tested in a pilot group of patients attending the emergency department to evaluate clarity, cultural appropriateness, and feasibility. The final questionnaire is provided as a Supplementary File (S1).

A total barrier score was computed by summing all item scores, yielding a possible range from 0 to 40. Higher scores indicate greater perceived barriers to pain medication use. Based on the distribution of scores and precedent in the literature, total scores were categorized into three levels: *low barriers* (0–13), *moderate barriers* (14–26), and *high barriers* (27–40). Subscales include fear of addiction, tolerance, side effects, fatalism, good patient role, and concern about distracting the doctor. Pain intensity was measured at presentation using the 0–10 Numerical Pain Rating Scale (NPRS), where 0 indicates no pain and 10 indicates worst possible pain. Pain severity was categorized as: 0–3 (mild), 4–6 (moderate), and 7–10 (severe) based on commonly used clinical cut‐offs. Participants with an NPRS score ≥ 4 were considered to have clinically significant pain (moderate to severe). This categorization has been utilized in prior clinical pain studies [17].

### 2.5 Inclusion and Exclusion Criteria

Inclusion in the study were patients aged 18 years and above, presenting to the ED with moderate to severe pain (≥ 4 on a 0–10 numeric pain rating scale), conscious and oriented to provide informed consent, able to communicate in English or the local language with assistance if necessary, and experiencing acute or sub‐acute pain, such as trauma, injury, or illness‐related pain (defined as non‐traumatic pain due to acute medical conditions, such as acute abdominal pain from appendicitis, biliary colic, renal colic, gastroenteritis, and inguinal hernia), and musculoskeletal pain associated with acute infection or inflammation. Pharmacological analgesia was the standard pain management strategy in this setting. The study did not include interventional pain procedures due to contextual practice patterns.

Excluded from the study were unconscious or critically ill patients and unable to provide informed consent or participate in an interview, those with cognitive impairment or psychiatric conditions that affect comprehension or response reliability, patients under the influence of alcohol or substances at the time of recruitment, patients with chronic pain conditions who are on long‐term opioid therapy, and patients who decline or withdraw consent.

### 2.6 Data Collection

Trained research assistants administered the questionnaire in English and local languages where needed. Demographic and clinical variables (age, gender, education, pain score, diagnosis, and type of treatment) were also collected. At presentation to the emergency room, pain was assessed using NPRS. After the first dosage of analgesic medication was administered in the emergency room, post‐treatment pain intensity was measured at a standard time point of 30 min (± 5 min). This post‐treatment timing was selected to capture the expected clinical response to pain management before additional interventions.

### 2.7 Informed Consent

Prior to participation and data collection, all eligible patients presenting with moderate to severe pain (≥ 4 on the Numeric Pain Rating Scale) at the emergency department were informed about the purpose, procedures, risks, and benefits of the study. They were advised that participation is entirely voluntary, and that refusal or withdrawal would not affect their access to medical care. Confidentiality was assured that data would be anonymized, securely stored, and used solely for research purposes. Written informed consent was given by the participants. The document was read aloud to individuals who could not read or write, and their thumbprints were used to express their assent. Ethical approval was obtained from the Institutional Review Board of the University of Cape Coast, reference number (UCCIRB/EXT/2025/024).

### 2.8 Data Analysis

Data were analyzed using STATA v14. Descriptive statistics summarized demographic data and barrier scores, including frequencies and means ( ± SD). Chi‐square tests and ANCOVA were used to assess associations. Associations between sociodemographic characteristics and perceived barriers to pain medication use were examined using Chi‐square tests. Adjusted residuals were used to determine which specific subgroup combinations contributed most strongly to statistically significant associations, with values ≥ 1.96 considered indicative of a significant contribution. Differences in post‐treatment pain scores between analgesic groups were explored using ANCOVA. A *p*‐value < 0.05 was considered statistically significant. All tests reported were two‐sided. The outcome variable derived from the adapted 8‐item barriers questionnaire scores ranged from 0 to 40, with higher scores indicating greater perceived barriers. Missing data were handled using a complete‐case approach in the analyses (proportion of missing data for key variables was low ( < 5%). Denominators vary due to missing responses for some variables; percentages are based on available data for each item.

## 3 Results

### 3.1 Demographic Characteristics of Study Participants

A total of 386 patients participated in the study. The mean age was 31 ± 11.4, with participants being predominantly females (52.6%), as compared to males (47.4%). The majority were identified as Christian (90.4%), while Muslims comprised 9.6%.

Regarding educational attainment, more than half (52.4%) had tertiary education, 26.2% had secondary education, 17% had completed primary education, while 4.3% had no formal education. On occupation, students represented the largest subgroup (34.2%), followed by those engaged in other occupations (27.5%), trading (19.4%), teaching (12.7%), and farming (6.2%), with details shown in Table 1.

**Table 1:** Frequency distribution of demographic characteristics of study participants using descriptive statistics (*N* = 386).

Variables	f	%
Age (mean ± SD) = 31 ± 11.4		
Gender		
Male	183	47.4
Female	203	52.6
Total	386	100
Religion		
Christian	339	90.4
Muslim	36	9.6
Total	375	100
Educational level		
Primary	63	17
Secondary	97	26.2
Tertiary	194	52.4
No formal education	16	4.3
Total	370	100
Occupation		
Student	132	34.2
Teaching	49	12.7
Trading	75	19.4
Farming	24	6.2
Others	106	27.5
Total	386	100

*Note:* Denominators vary due to missing responses for some variables; percentages are based on available data for each item.

### 3.2 Sociodemographic Factors and Reported Barriers to Pain Medication Use

Associations between sociodemographic characteristics and levels of perceived barriers to pain medication use were evaluated using Chi‐square tests with adjusted residuals to identify cells contributing to significant associations. Gender, occupation, and educational level were significantly associated with barrier categories (*p* < 0.001 for all), whereas religion was not (*p* = 0.17). With regards to gender, male participants were more likely to report moderate barriers (56.7%), with fewer reporting low barriers (43.6%) and none reporting high barriers. In contrast, among the female participants (56.4%) exhibited low barriers, whereas and (43.4%) indicated moderate barriers, with all high barrier cases (27 individuals) being female. Adjusted residuals indicate that females contributed substantially to the high‐barrier category (residual = +5.12), whereas males contributed less than expected to the high‐barrier category (residual = –5.12).

Concerning occupation, students comprised a large proportion of the moderate (49.3%), and high (59.3%) barrier scores, respectively. Those in the trading category had somewhat more high barrier reports (40.7%) compared to moderate or low barriers. On the other hand, teachers and those in farming or other occupations generally reported higher proportions of low or moderate barriers, with no high barrier cases observed among them. Adjusted residuals suggest that student status was strongly associated with moderate barrier reporting (residual = +6.60), whereas “other” occupations were more likely than expected to report low barriers (residual = +5.62). Regarding religious status, the distribution of Christian and Muslim participants across low, moderate, and high barrier levels did not differ substantially from expected counts, as indicated by adjusted residuals close to 0.

However, participants with tertiary education were more likely than expected to report moderate (62%) and high barriers (100% of high barrier cases), with adjusted residuals showing a strong contribution to these categories. Those with primary and secondary education were more likely to report low barriers, with significant positive adjusted residuals in the low barrier cells. Participants with no formal education were exclusively in the low barrier category in this sample, although the group was small, with details shown in Table 2.

**Table 2:** Association between sociodemographic factors and reported barriers to pain management using Chi‐square test with adjusted residuals (*N* = 386).

Variables	Low *n* (%)	Moderate *n* (%)	High *n* (%)	Adj. residual (L/M/H)	*p* value
Gender					*p* < 0.001
Male	68 (43.6)	115 (56.7)	0	−1.24/3.8/−5.12	
Female	88 (56.4)	88 (43.4)	27 (100)	1.24/3.83/5.12	
Occupation					*p* < 0.001
Student	16 (10.3)	100 (49.3)	16 (59.3)	−8.17/6.6/2.85	
Teaching	24 (15.3)	25 (12.3)	0	1.31/−0.24/−2.1	
Trading	37 (23.7)	27 (13.3)	11 (40.7)	1.75/−3.21/2.9	
Farming	12 (7.7)	12 (5.9)	0	0.99/−0.3/−1.38	
Other	67 (43)	39 (19.2)	0	5.62/−3.83/−3.3	
Religion					*p* = 0.173
Christian	114 (92.3)	179 (88.2)	16 (100)	1.1/−1.59/1.33	
Muslim	12 (7.7)	24 (11.8)	0	−1.06/1.59/−1.3	
Education level					*p* < 0.001
Primary	40 (25.6)	23 (12.3)	0	3.76/−2.45/−2.4	
Secondary	49 (31.4)	48 (25.7)	0	1.94/−0.24/−3.2	
Tertiary	51 (32.7)	116 (62)	27 (100)	−6.49/3.3/5.14	
No formal education	16 (10.3)	0	0	4.8/−4.13/−1.15	

Abbreviations: Adj., adjusted; H, high; L, low; M, moderate; *n*, frequency.

### 3.3 Perceived Barriers to Pain Medication Use

Participants' responses to eight selected items from the Barriers Questionnaire provided insight into prevalent beliefs that may impact pain medication use. Each item was scored on a Likert scale ranging from 0 (do not agree at all) to 5 (agree very much), with details as shown in Table 3.

**Table 3:** Analysis of perceived barriers to pain management using descriptive statistics.

Perceived barriers	f	Mean	SD
Pain meds cannot control pain	386	1.34	1.69
People get addicted to pain meds	375	2.25	2.04
Good patients avoid talking about pain	386	1.25	1.68
Experience of pain means illness has become worse	375	3.77	1.32
It is easy to bear pain than side effects of pain meds	354	1.78	1.85
Pain medications should be saved for future use	386	2.37	2.16
Pain builds character	386	1.08	1.67
Pain could distract doctor from curing residents' problem	373	1.31	1.60

*Note:* Frequencies vary due to missing responses for some variables; percentages are based on available data for each item.

Abbreviations: f, frequency; med, medication; SD, standard deviation.

The most strongly endorsed barrier was the belief that “experiencing pain means the illness has become worse” (mean = 3.77, SD = 1.32), indicating that a large proportion of patients associate pain with disease progression.

Moderate concern was observed regarding the fear that “people get addicted to pain medications” (mean = 2.25, SD = 2.04), and the belief that “pain medications should be saved for future use” (mean = 2.37, SD = 2.16).

Other barriers such as “pain medications cannot control pain” (mean = 1.34), “it is easier to bear pain than side effects of medication” (mean = 1.78), and “pain could distract the doctor from curing the real problem” (mean = 1.31) were less strongly endorsed, indicating that the majority of respondents trusted the efficacy of pain medications and were not significantly influenced by fear of side effects or miscommunication.

Notably, cultural or moral beliefs about pain were slightly endorsed. For instance, only a small fraction agreed with the statement “pain builds character,” and few believed that “good patients avoid talking about pain.”

### 3.4 Association Between Analgesic Type and Post‐Treatment Pain Score

Table 4 describes the association between the type of analgesic used and the post‐treatment pain scores. An analysis of covariance (ANCOVA) was performed to examine the association between analgesic type and post‐treatment pain score while adjusting for pre‐treatment pain score and type of diagnosis. The overall model was statistically significant (*F* (9, 364) = 69.95, *p* < 0.001) and explained approximately 63% of the variance in post‐treatment pain scores (adjusted *R*² = 0.625).

**Table 4:** Analysis of covariance (ANCOVA) examining the association between analgesic type and post‐treatment pain score, adjusting for covariates (*n* = 374).

Variable	*β* (coefficient)	Standard error	*t* value	*p* value	95% CI
Diclofenac (ref)					
Tramadol	−0.21	0.14	−1.44	0.15	−0.49, 0.08
Pethidine	4.16	0.38	10.98	< 0.001	3.42, 4.91
Morphine	−2.34	0.27	−8.53	< 0.001	−2.88, −1.80
Buscopan	−0.91	0.25	−3.64	< 0.001	−1.40, −0.42
Diclofenac + Tramadol	0.97	0.34	2.84	0.005	0.30, 1.65
Diclofenac + Buscopan	−0.64	0.22	−2.95	0.003	−1.07, −0.21
Tramadol + Buscopan	−3.84	0.38	−10.13	< 0.001	−4.58, −3.09
Covariates					
Type of diagnosis (p18)	0.17	0.02	8.11	< 0.001	0.13, 0.22
Pre‐treatment pain score (p9)	0.49	0.05	10.63	< 0.001	0.40, 0.58

*Note:* Model statistics: *F* (9, 364) = 69.95, *p* < 0.001; Adjusted *R*² = 0.625; Root MSE = 1.09.

Abbreviations: *χ*², Chi‐square statistic; CI, confidence interval; df, degrees of freedom; *β*, regression coefficient (slope).

After adjustment for covariates, the type of analgesic administered showed a significant overall association with post‐treatment pain (partial *F* = 64.86, *p* < 0.001). Compared with the diclofenac, pethidine and diclofenac + tramadol were associated with significantly higher post‐treatment pain scores, whereas morphine, buscopan, diclofenac + buscopan, and tramadol + buscopan were associated with significantly lower post‐treatment pain scores. Tramadol did not differ significantly when compared to diclofenac.

Both covariates independently predicted post‐treatment pain. Pre‐treatment pain score was a strong positive predictor (*β* = 0.49, *p* < 0.001). Type of diagnosis was also significantly associated with post‐treatment pain (*β* = 0.17, *p* < 0.001), after adjusting for baseline pain and the type of analgesic used.

## 4 Discussion

This study assessed barriers to pain management and outcome in an emergency care setting in Ghana, and explored factors associated with these barriers, including demographic characteristics, clinical presentation, and pain management outcomes. The findings highlight important insights into how sociodemographic factors, analgesic prescribing patterns, and attitudinal beliefs may influence pain management outcomes. Post‐treatment pain outcomes varied by analgesic category even after adjusting for baseline pain severity and diagnosis.

The findings revealed that most participants demonstrated high perceived barriers, indicating prevalent misconceptions and fears regarding pain medication, and consistent with recent research in various contexts [15, 18]. In low‐resource environments similar to ours, barriers such as limited health literacy, and cultural fatalism have been identified as key factors undermining effective pain management [19, 20, 21]. Our results highlight the critical need for culturally appropriate interventions, such as targeted education programs, provider training, and improved communication to address and diminish these widespread barriers in acute care settings.

The analysis of perceived barriers revealed that the most strongly endorsed belief was that “experiencing pain means the illness has become worse,” a fatalistic interpretation that has been reported in previous studies as a psychological barrier to optimal pain control [8, 9]. This belief may increase patients' anxiety and influence their perception of pain severity, regardless of clinical indicators. Moderate concerns were noted regarding addiction and the belief that pain medications should be reserved for future use. These findings are consistent with prior research indicating that fears of addiction and tolerance often lead to delayed or underutilized analgesic therapy, particularly in low‐ and middle‐income countries [13, 22, 23]. Such beliefs may contribute to the ongoing problem of inadequate pain management in emergency care. On the contrary, cultural or moral beliefs about pain were slightly endorsed, as few believed that “good patients avoid talking about pain.” This suggests that most patients were open to discussing pain and did not associate endurance with personal strength. Overall, while fatalistic beliefs and concerns about addiction were evident, most participants did not endorse cultural or communicative barriers, reflecting a generally favorable attitude toward expressing pain and receiving appropriate treatment.

Our analysis revealed significant associations between sociodemographic characteristics and perceived barriers to pain medication use. Beyond overall Chi‐square significance, adjusted residuals clarified which subgroups contributed disproportionately to these patterns. Female participants were significantly more likely than expected to report high barriers, whereas males were underrepresented in this category, suggesting a gendered dimension to pain management perceptions. This finding aligns with literature showing systematic differences in how pain is experienced, communicated, and treated between women and men. Studies have demonstrated that women often report higher pain intensity and exhibit greater hesitancy toward analgesic use, and that they may receive less adequate pain management in clinical settings compared with men, reflecting both biological and sociocultural factors influencing pain perceptions and treatment decisions [24]. Additionally, broader literature has documented gender biases in pain assessment and management, where women's pain reports are sometimes underestimated or dismissed by clinicians, potentially reinforcing hesitancy and barriers to effective analgesic use [25].

Among occupational groups, students showed higher than expected counts in the moderate and high barrier categories, which may reflect distinct health beliefs, healthcare literacy, or prior experiences with pain and healthcare systems. Young adults and students are often less experienced with formal healthcare and may hold greater misconceptions about medications or fear adverse effects, beliefs that have been shown to influence medication hesitancy in other healthcare context [26].

Interestingly, participants with tertiary education were overrepresented in higher barrier categories, indicating that higher formal education does not necessarily translate to lower perceived barriers and may even reflect concerns about medication safety, side effects, or long‐term impacts [27]. This pattern is consistent with research on medication attitudes beyond pain, where higher education is associated with more critical appraisal of medications and heightened awareness of potential risks [28], which can paradoxically increase perceived barriers to use.

These insights underscore the importance of adjusted residuals for interpreting associations in categorical data and highlight key subgroups for targeted interventions, such as gender‐sensitive pain communication strategies, educational interventions that address misconceptions among more educated patients, and tailored support for younger or student populations.

Our adjusted ANCOVA findings indicate that the type of analgesic administered was significantly associated with post‐treatment pain scores, even after accounting for baseline pain severity and diagnosis. Specifically, analgesic regimens such as morphine, buscopan‐based combinations, and tramadol with buscopan were associated with lower post‐treatment pain compared with diclofenac alone, whereas pethidine and the diclofenac–tramadol combination were associated with higher residual pain. This pattern suggests that opioid‐based and multimodal analgesic strategies may offer superior pain control in acute pain contexts. Evidence from randomized and observational studies supports the concept that multimodal analgesia, which combines agents targeting different pain pathways, improves analgesic outcomes and reduces reliance on single agents compared with traditional opioid‐only regimens, with corresponding improvements in pain scores and reduced opioid‐related adverse effects [29].

Tramadol, while often effective for moderate pain, has been shown to have variable analgesic potency relative to other opioids, and its effectiveness may be modest when used in isolation, aligning with our finding that tramadol alone did not significantly differ from diclofenac [30]. Pethidine (meperidine) has historically been used for moderate to severe pain but has comparable efficacy relative to type of condition, which may contribute to its association with higher post‐treatment pain in our data [31]. Moreover, the strong positive associations of baseline pain with post‐treatment scores underscore the importance of early and adequate pain control, a finding consistent with clinical reports on postoperative pain pathways [32]. These findings underscore how analgesic performance must be interpreted in light of various factors, including attitudes, beliefs, and clinical procedure [33]. Collectively, our results may help inform considerations for tailored analgesic selection and the potential role of multimodal strategies to optimize pain relief in clinical practice. The current study, however, improves understanding of how patient beliefs and misconceptions about pain and analgesics may hinder effective pain management in resource‐limited emergency settings, highlighting a critical but under‐studied barrier to rational drug use.

Although our study focused exclusively on pharmacological analgesia, it is important to acknowledge that interventional pain strategies such as peripheral nerve blocks are increasingly recognized as effective options in emergency care. Evidence shows that ultrasound‐guided nerve blocks, including fascia iliaca and femoral nerve blocks, can provide rapid and sustained pain relief for patients with acute conditions such as hip and femoral neck fractures, while also reducing opioid requirements and associated side effects [34]. Major emergency medicine societies support the use of nerve blocks and other regional anesthesia techniques as part of multimodal pain management in emergency settings, and training in these approaches is increasingly incorporated into emergency medicine education [35, 36]. However, barriers to implementation remain, especially in low‐ and middle‐income settings, where lack of training, limited access to equipment, and workforce constraints impede the routine use of nerve blocks in emergency departments. For example, surveys in African emergency and trauma centers suggest that although clinicians recognize the value of nerve blocks, they are under‐utilized due to gaps in knowledge, skill, and resources [37]. Given our findings on patient‐reported barriers to analgesic use, future research could explore patients' attitudes and acceptability of interventional pain techniques in our setting, as cultural perceptions and structural factors may further influence the uptake of these advanced pain management options.

### 4.1 Strengths and Limitations

This study presents several strengths that enhance the credibility and relevance of its findings. The conduction of the research in a real‐world emergency department ensured that the data reflects patient experiences and clinical practice conditions. Moreover, the use of adapted items from the validated Barriers Questionnaire (BQ) provided a structured and conceptually sound approach to assessing patient attitudes toward pain and analgesic use. Additionally, the integration of clinical data such as pain scores and medication types, with psychosocial variables provided a comprehensive perspective on the multidimensional factors influencing pain management. The major limitation of the study is its cross‐sectional nature, which limits the ability to establish causal relationships or monitor changes over time. Although we used statistical adjustments (ANCOVA) to control baseline pain and diagnosis in post‐treatment pain comparisons, residual confounding by unmeasured factors such as prior pain experience, comorbidities, and individual health beliefs may still influence the observed associations. Thus, conclusions regarding the relative effectiveness of different analgesics should be regarded as associative rather than causal. Also, self‐reported data on beliefs are inherently subject to recall bias and social desirability effects, particularly in an emergency care setting where patients may be in distress. Pain perception is subjective and influenced by numerous biopsychosocial factors, including mood, prior pain experiences, and cultural norms, which we did not assess in this study. Additionally, this study was conducted in a single emergency department in a specific healthcare context, which may limit the generalizability of the findings to other settings with different patient populations and clinical protocols.

## 5 Conclusion

Patient misconceptions about pain and its treatment persist in emergency care settings and may impact both pain reporting and medication adherence. Overall, the study underscores the multifaceted nature of pain management, where clinical effectiveness is influenced not only by drug choice but also by patient beliefs, provider practices, and systemic constraints. This study, therefore, underscores the necessity of a comprehensive approach to improving pain management. Education strategies that address misconceptions about addiction, medication timing, and pain expression could significantly improve pain outcomes. While our findings advance understanding of patient‐reported barriers and early post‐treatment pain outcomes in an emergency setting, they should be interpreted within the context of the study's methodological constraints. Future research will be essential to develop effective, evidence‐based interventions that improve pain management across diverse patient populations, and to explore longitudinal designs to assess changes in pain perception over time.

## Author Contributions

**Stephen Mensah Arhin:** conceptualization, methodology, writing – original draft, writing – review and editing, supervision, funding acquisition. **Isaac Tabiri Henneh:** conceptualization, formal analysis, writing – review and editing. **Joseph Arhin:** methodology, formal analysis, writing – review and editing. **James Kojo Prah:** supervision, formal analysis, writing – review and editing. **Emmanuel Nkrumah:** formal analysis, writing – review and editing. **Oluwayemisi Esther Ekor:** methodology, writing – review and editing. **Kwesi Boadu Mensah:** methodology, supervision, writing – review and editing. **Martins Ekor:** formal analysis, supervision, writing – review and editing, funding acquisition. All authors have read and approved the final version of the manuscript. Stephen Mensah Arhin had full access to all the data in this study and takes complete responsibility for the integrity of the data and the accuracy of the data analysis.

## Funding

The authors received no specific funding for this work.

## Disclosure

The lead author Stephen Mensah Arhin affirms that this manuscript is an honest, accurate, and transparent account of the study being reported; that no important aspects of the study have been omitted; and that any discrepancies from the study as planned (and, if relevant, registered) have been explained.

## Ethics Statement

Ethical approval for the study was obtained from the Institutional Review Board of the University of Cape Coast, with reference number UCCIRB/EXT/2025/024. Written informed consent was obtained from the study participants.

## Conflicts of Interest

The authors declare no conflicts of interest.

## Supporting information

S1 SuppInfo.

## Data Availability

The data that support the findings of this study are available from the corresponding author upon reasonable request.
