# Supplementary material for: Pain Experience and Patient‐Reported Barriers to Analgesic Use in Emergency Care in Ghana: A Cross‐Sectional Study
Source: Health Sci Rep. 2026 Mar 19;9(3):e72135. doi: 10.1002/hsr2.72135 (PMC13098095; doi:10.1002/hsr2.72135)
Supplement: Supplementary file 1 — S1 SuppInfo. [file HSR2-9-e72135-s001.docx]

**Questionnaire**

This questionnaire is designed to assess beliefs and concerns that may interfere with effective pain management. Please indicate how much you agree or disagree with the following statements, using the scale provided. This research is for academic purposes only.

**Section A:** Socio-demographic characteristics of participants

**Age:**

**Sex:** [ ] Male [ ] Female

**Occupation:** [ ] Student [ ] Teaching [ ] Trading [ ] Farming [ ] Other

**Education level:** [ ] Primary [ ] Secondary [ ] Tertiary [ ] No formal education

**Religion:** [ ] Christian [ ] Christian [ ] Other

**Section B:** Beliefs about pain management

**Response scale:** 0= Do not agree at all**;** 1= Do not agree**;** 2= Do not agree somehow**;** 3= Agree somehow**;** 4= Agreed**;** 5=Agreed very much

| **Item** | **0** | **1** | **2** | **3** | **4** | **5** |
| --- | --- | --- | --- | --- | --- | --- |
| Pain medications cannot control pain |  |  |  |  |  |  |
| People get addicted to pain medications |  |  |  |  |  |  |
| Good patients avoid talking about pain |  |  |  |  |  |  |
| Experience of pain means illness has become worse |  |  |  |  |  |  |
| Is easier to bear pain than side effects of pain medications |  |  |  |  |  |  |
| Pain medications should be saved |  |  |  |  |  |  |
| Pain builds character |  |  |  |  |  |  |
| Pain could distract doctor from curing residents’ problem |  |  |  |  |  |  |

**Section C: Pain assessment (Using the numerical pain scale, ranged from 0-10, where 0, means no pain, and 10 means very severe pain).**

Pain score before treatment:

Pain score after treatment:

**Diagnosis/Indications:**

**Type of Treatment (s) given:** …………………………………………………………………………………
